# Grain size dependence of dielectric relaxation in cerium oxide as high-*k* layer

**DOI:** 10.1186/1556-276X-8-172

**Published:** 2013-04-15

**Authors:** Chun Zhao, Ce Zhou Zhao, Matthew Werner, Steve Taylor, Paul Chalker, Peter King

**Affiliations:** 1 Department of Electrical Engineering and Electronics, University of Liverpool, Liverpool, L69 3GJ, UK; 2 Department of Electrical and Electronic Engineering, Xi'an Jiaotong-Liverpool University, Suzhou, Jiangsu, 215123, China; 3 School of Engineering, Center for Materials and Structures, University of Liverpool, Liverpool, L69 3GH, UK; 4 Present address: Nanoco Technologies Ltd, 46 Grafton Street, Manchester, M13 9NT, UK

**Keywords:** Cerium oxide, High-*k*, Grain size, Dielectric relaxation

## Abstract

Cerium oxide (CeO_2_) thin films used liquid injection atomic layer deposition (ALD) for deposition and ALD procedures were run at substrate temperatures of 150°C, 200°C, 250°C, 300°C, and 350°C, respectively. CeO_2_ were grown on *n*-Si(100) wafers. Variations in the grain sizes of the samples are governed by the deposition temperature and have been estimated using Scherrer analysis of the X-ray diffraction patterns. The changing grain size correlates with the changes seen in the Raman spectrum. Strong frequency dispersion is found in the capacitance-voltage measurement. Normalized dielectric constant measurement is quantitatively utilized to characterize the dielectric constant variation. The relationship extracted between grain size and dielectric relaxation for CeO_2_ suggests that tuning properties for improved frequency dispersion can be achieved by controlling the grain size, hence the strain at the nanoscale dimensions.

## Background

Recently, cerium oxide (CeO_2_) is proposed as a possible gate dielectric material in metal-oxide-semiconductor and memory devices for next generation devices [1,2]. This is because CeO_2_ can be epitaxially grown on a Si (111) surface [3] and also because its high ability for oxygen storage makes CeO_2_ one of the most important automobile exhaust catalysts [4]. CeO_2_ has a high dielectric constant [5,6] and may be used as a high-*k* gate dielectric to suppress gate leakage current. CeO_2_ has also been added to HfO_2_ in order to stabilize the high-*k* cubic and tetragonal phases for potential applications in sub-32-nm-node complementary metal oxide semiconductor (CMOS) devices [7,8]. In terms of microelectronic applications, atomic layer deposition (ALD) is the most attractive technique for the deposition of CeO_2_. This is due to its ability to deposit large areas of high-uniformity thin films, good doping control, and superior conformal step coverage on highly non-planar substrates [9]. In ALD, metal alkoxides have the major advantage of high reactivity with H_2_O, thus avoiding the formation of a low-permittivity interfacial layer during the ALD of high-*k* dielectrics [7].

**Figure 1:**
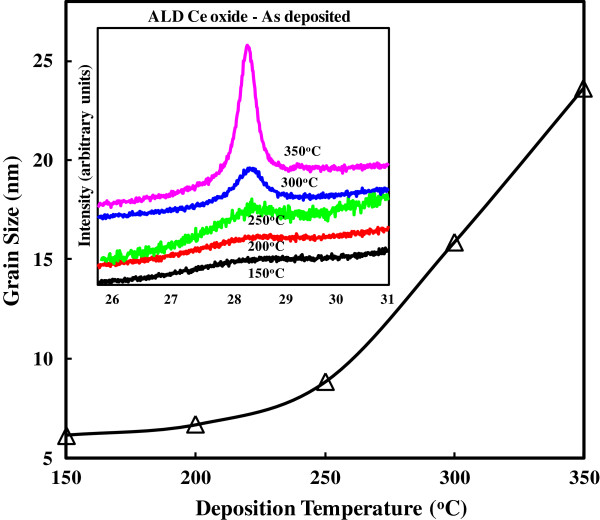
**Grain sizes for as**-**deposited CeO**_**2 **_**samples under different deposition temperatures (150°C**, **200°C**, **250°C**, **300°C, and 350°C).** XRD patterns are shown in the inset. Grain sizes (extracted from XRD data) increased following the increasing deposition temperatures.

**Figure 2:**
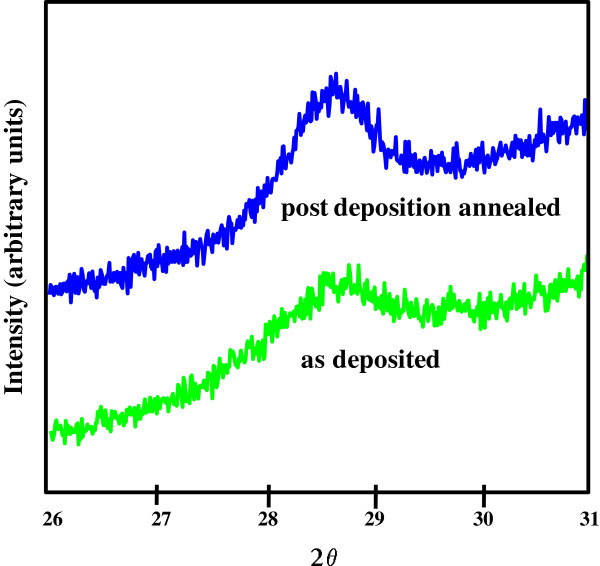
**XRD patterns for the 250°C samples (green for the as**-**deposited and blue for the post-deposition annealing).** The grain size of the annealed sample (9.55 nm) increased compared to the as-deposited sample (8.83 nm), which suggests that post-deposition annealing in vacuum causes an increase in the size of the crystalline grains.

**Figure 3:**
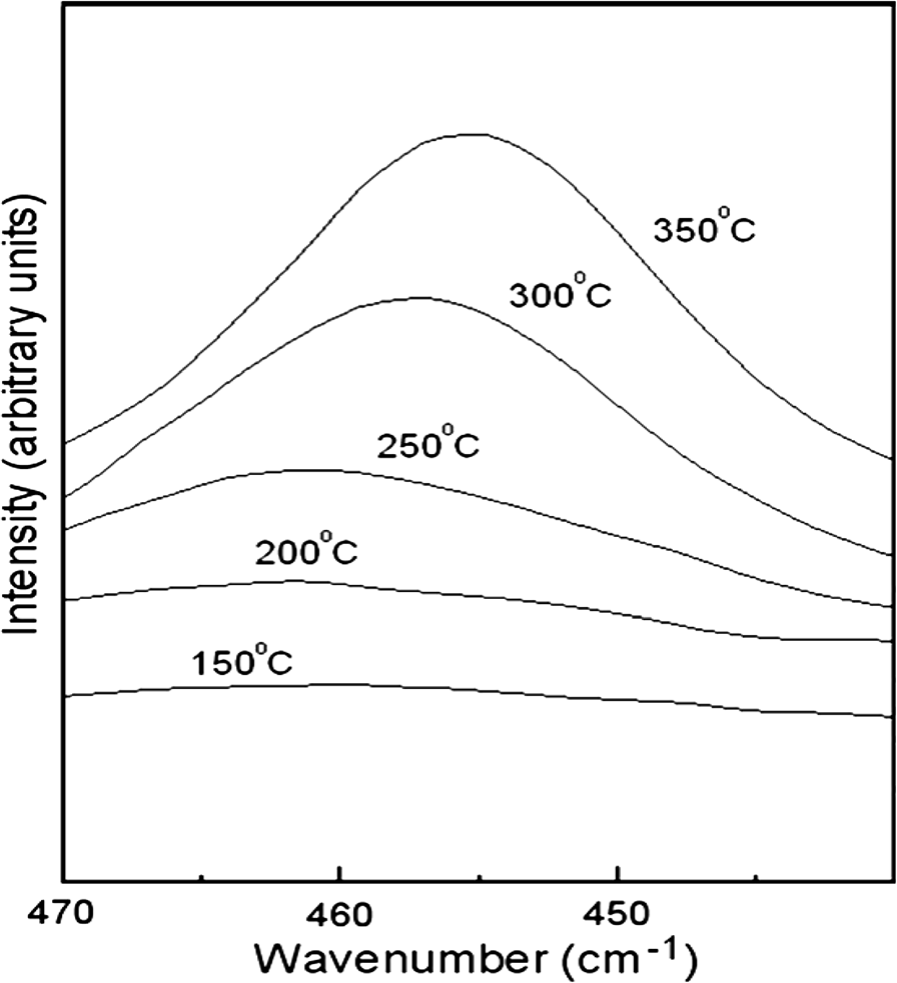
**Raman spectrum of CeO**_**2 **_**samples deposited under different temperatures (150°C**, **200°C**, **250°C**, **300°C, and 350°C).** Raman spectrum results are consistent with XRD data (inset of Figure 1): larger grain sizes were observed as the deposition temperature increases.

**Figure 4:**
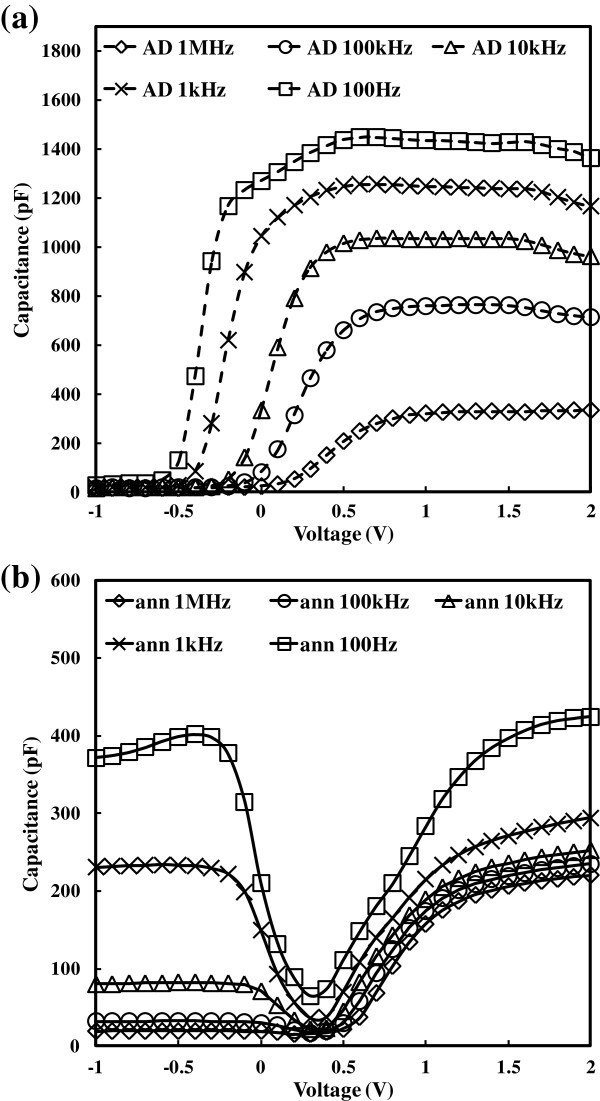
**Capacitance**-**voltage* (C***-***V) *measurements of the as**-**deposited (AD) and the annealed (ann) samples under different frequencies.** Frequencies: 100 Hz, 1 kHz, 10 kHz, 100 kHz, and 1 MHz. Remarkable frequency dispersions on *C*-*V* curves are observed for both the as-deposited **(a)** and the annealed samples **(b)**. Compared to the as-deposited samples, the annealed samples show pronounced accumulation capacitance reduction. The most important effect of annealing is related to weakened accumulation capacitance and hence reduced *k*-value.

**Figure 5:**
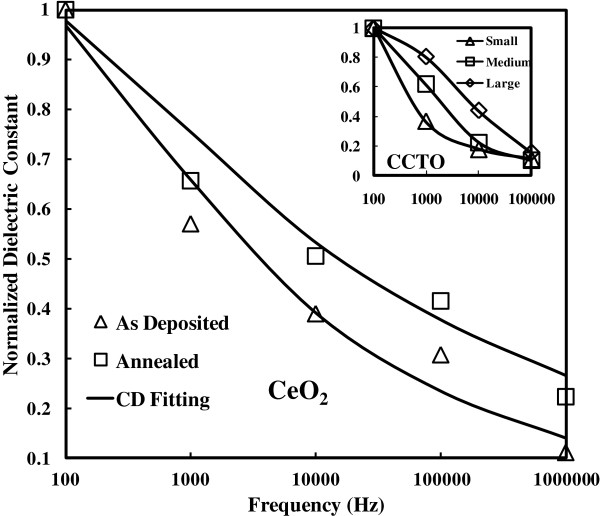
**Normalized dielectric constants for as**-**deposited and annealed samples under different frequencies.** Frequencies: 100Hz, 1 kHz, 10 kHz, 100 kHz, and 1 MHz. The grain size of the annealed sample (9.55 nm) is larger than the as-deposited sample (8.83 nm), of which the grain size values are extracted from the XRD data (Figure 2). It is clear that dielectric relaxation for the as-deposited sample (triangle symbol) is much worse than that of the annealed one (square symbol). The Cole-Davidson fitting data are represented by solid lines. Normalized dielectric constants for the CaCu_3_TiO_12_ (CCTO) samples [18] under different frequencies (100Hz, 1 kHz, 10 kHz, and 100 kHz) are given in the inset as supporting evidence. Similar to CeO_2_, dielectric relaxation for the medium-grain-size CCTO sample is superior to the small sample within the entire frequency range. Moreover, the large-grain-size sample is better than the medium one in terms of dielectric relaxation. Therefore, grain size makes a significant impact on dielectric relaxation.

**Figure 6:**
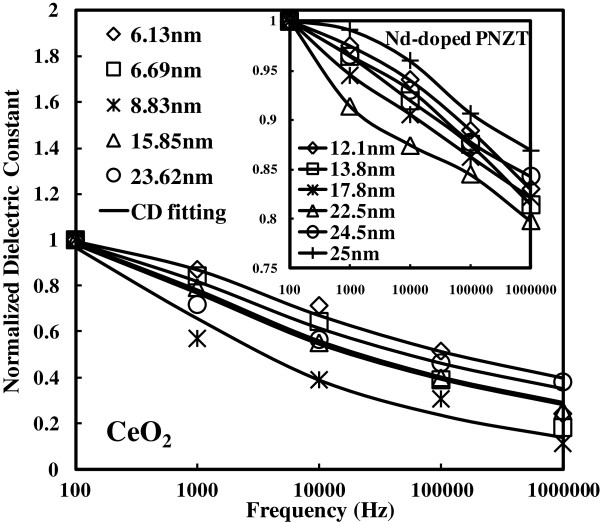
**Normalized dielectric constants for as**-**deposited samples under different frequencies.** Frequencies: 100 Hz, 1 kHz, 10 kHz, 100 kHz, and 1 MHz. The grain size value for the samples of the different deposition temperatures (Figure 1) is denoted with respective symbols (diamond, square, star, triangle, and round). The Cole-Davidson fitting for each curve is represented by a solid line. The sample of 8.83 nm has the most severe dielectric relaxation. However, in comparing the samples of 6.13 and 23.62 nm, the larger-grain-size sample is proved to have better performance on dielectric relaxation. Similarly, normalized dielectric constants for the Nd-doped PNZT samples [19] are shown in the inset under various frequencies (100 Hz, 1 kHz, 10 kHz, 100 kHz, and 1 MHz) as supporting evidence. The grain size value for each sample is denoted with respective symbols (diamond, square, star, triangle, round, and cross). It is obvious that the deteriorative degree of dielectric relaxation increases from 12.1 nm, reaches the peak at 22.5 nm, and then is relieved much to a better situation. The last sample with the grain size of 25 nm is shown to have dielectric relaxation superior to the sample of 12.1 nm.

**Figure 7:**
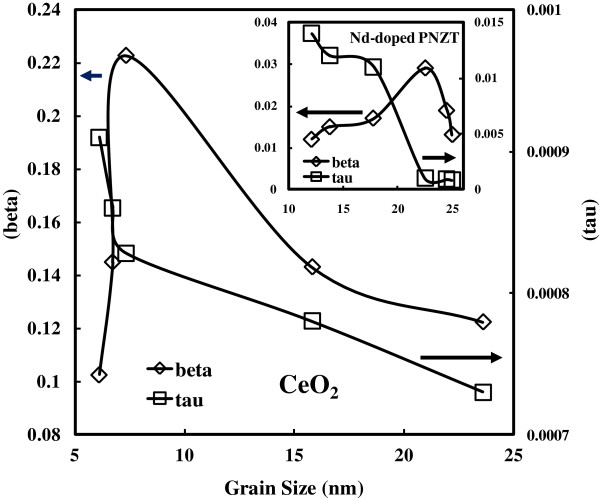
**Cole**-**Davidson fitting parameters *β *and *τ *for as**-**deposited CeO**_**2 **_**samples with different grain sizes.** It is clear that the trend of beta increases from 6.13 nm, peaks at 8.83 nm with the beta value of 0.21, and then descends. The trend of tau decreases from 6.13 to 23.62 nm. Therefore, the trend of beta is consistent with the deteriorative degree of dielectric relaxation. The inset shows the relationship between the Cole-Davidson fitting parameters (beta and tau) and the grain size values for the Nd-doped PNZT samples (data from [19]). The trend of beta (the deteriorative degree of dielectric relaxation) rises from 12.1 nm, peaks at 22.5 nm with the beta value of 0.03, and then declines within the range of 22.5 to 25 nm. The trend of tau decreases from 12.1 to 25 nm accordingly, similar to the CeO_2_ samples.

It is well known that the optical and electrical properties of CeO_2_ are highly dependent on the surface and interface structure, morphology, and chemistry [10], which in turn is controlled by the fabrication technique and growth conditions [11]. The ability to tailor the properties so as to optimize performance requires a detailed understanding of the relationship between electronic and geometric structures, particularly at nanoscale dimensions, of CeO_2_. CeO_2_ readily crystallizes in the fluorite form, but control over the grain size formed is important due to the effect of grain boundary density on properties like ionic conductivity and dielectric response [12]. Moreover, the intrinsic frequency dispersion (dielectric relaxation) studies [13,14] have also been found to be relevant to grain size of the samples, especially those dealing with nanostructured materials.

In this paper, CeO_2_ is prepared by ALD under different deposition temperatures. The grain size of the samples is determined respectively by the fabrication technique and growth conditions. The focus of the present work is, therefore, on elucidating grain size effects on the electrical properties of CeO_2_. An interesting correlation between grain size and dielectric relaxation, which provides a reference to tailor the properties and performance of CeO_2_ as a high-*k* thin film, has been presented and discussed in the paper.

## Methods

The CeO_2_ thin films were deposited by liquid injection ALD via a modified Aixtron AIX 200FE AVD reactor (Herzogenrath, Germany) fitted with a liquid injector system. The precursor was a 0.05-M solution of [Ce(mmp)4] (SAFC Hitech Ltd, Dorset, England, UK) in toluene [9], and the source of oxygen was deionized water. ALD procedures were run at substrate temperatures of 150°C, 200°C, 250°C, 300°C, and 350°C, respectively. The evaporator temperature was 100°C, and the reactor pressure was 1 mbar. The CeO_2_ thin films were grown on *n*-Si(100) wafers. Argon carrier gas flow was performed with 100 cm^3^/min. The flow of [Ce(mmp)_4_]/purge/H_2_O/purge was 2:2:0.5:3.5 s, and the number of growth cycles was 300.

For physical characterization, X-ray diffraction (XRD) was achieved using a Rigaku miniflex diffractometer (Shibuya-ku, Japan) with CuK_α_ radiation (0.154051 nm, 40 kV, 50 mA), spanning a 2θ range of 20° to 50° at a scan rate of 0.01°/min. Raman spectra were obtained with a Jobin-Yvon LabRam HR consisting of a confocal microscope coupled to a single grating spectrometer equipped with a notch filter and a charge-coupled device camera detector. Raman measurements were performed using a confocal aperture of 300 μm to limit the light to improve the quality of signal. The measurements were spanned with 150 to 1,500/cm of four accumulations, and the exposure time was 30 s. All of the spectra were observed using an incident wavelength of 325 nm from a He-Cd laser. To determine the electrical characteristics of the CeO_2_ samples, capacitance-voltage (*C*-*V*) measurements were implemented using an Agilent E4980A precision LCR meter (Santa Clara, CA, USA). Gold contacts were deposited with an area of 4.5 × 10^-4^ cm^2^, and aluminum was deposited onto the backside of the silicon substrate.

## Results and discussion

XRD diffraction patterns for the as-deposited CeO_2_ thin films at 150°C, 200°C, 250°C, 300°C, and 350°C, respectively, are shown in the inset of Figure 1. Diffraction scans with a slower scan speed were performed in the region of the peak to obtain full width at half-maximum data (the most distinct diffraction peak). XRD results show crystalline diffraction features for all deposition temperatures. The grain size value is obtained using the Scherrer formula [15] based on the XRD data (Figure 1). The measurements performed have the grain size changing from 6.14 nm for the 150°C sample to 23.62 nm for the 350°C sample. For the 200°C, 250°C, and 300°C samples, the grain sizes are 6.69, 8.83, and 15.86 nm, respectively. There is a clear trend that the grain size increases with increasing deposition temperatures. The proposed explanation is most likely due to the high deposition temperature contributing to the settling of the atoms to their lattice sites. Post-deposition annealing (PDA) was operated on the 250°C as-deposited samples in vacuum at 800°C for 5 min. Figure 2 shows the XRD diffraction patterns for the as-deposited and annealed samples, respectively. The grain size of the annealed sample (9.55 nm) is bigger than the original sample (8.83 nm), which suggested that PDA in vacuum causes an increase in the size of the crystalline grains. The same phenomenon is also observed in the 150°C as-deposited samples after PDA.

Raman spectra of the same CeO_2_ thin films deposited at five substrate temperatures (150°C, 200°C, 250°C, 300°C, and 350°C) are shown in Figure 3. The data show a distinct shift on the intensity axis following the increased deposition temperature. The first-order triply degenerate mode is the mode at approximately 465/cm associated with the fluorite crystal structure. The measurement presented confirms that the crystalline phase is cubic. A clear shift to a higher wave number together with a broadening of the band with decreasing temperature is observed. Decreased phonon lifetime with smaller grain size is the main reason for the broadening effect. The peak shift to a higher wave number is due to a releasing of the chemical bonds for smaller grain size at the lower deposition temperature. Comparing the five Raman spectra, their intensities relatively decrease as the grain size decreases [16]. Consequently, Raman results are correlated to XRD data: Raman spectral data and XRD results share the same trend that larger grain sizes are formed as the deposition temperature increases.

*C*-*V* measurements are used to characterize frequency dispersion [17] and to obtain permittivity of the CeO_2_ thin films. A typical set of *C*-*V* characteristics of the as-deposited (dashed line) under different frequencies (100 Hz, 1 kHz, 10 kHz, 100 kHz, and 1 MHz) is shown in Figure 4 for the sample deposited at 250°C. *C*-*V* measurements are carried out from strong inversion (-1 V) toward strong accumulation (2 V). Noticeable frequency dispersion on *C*-*V* curves is observed. Frequency dispersion in *C*-*V* or capacitance-frequency measurements are categorized into two parts: extrinsic and intrinsic. Extrinsic frequency dispersion includes (1) parasitic effect, (2) lossy interfacial layer effect, (3) surface roughness effect, (4) polysilicon depletion effect, and (5) quantum mechanical effect. For part 1 of the extrinsic frequency dispersion, parasitic effects in MOS devices contain parasitic resistances and capacitances such as bulk series resistances, contacts (including contact between the MOS capacitor and probe station), cables, and many other parasitic effects. The parasitic effects can simply be minimized by using suitable cables and also by depositing an aluminum thin film at the back of a large-area silicon substrate. For the cerium oxide samples, the aluminum back contact and substrate area is approximately 2 × 2 cm^2^. Concerning part 2, the existence of extrinsic frequency dispersion in some high-*k* materials (LaAlO_3_) is mainly due to the effect of the lossy interfacial layer between the high-*k* thin film and silicon substrate on the MOS capacitor. Relative thicker thickness of the high-*k* thin film than the interfacial layer significantly prevented frequency dispersion. For the cerium oxide samples, the high-*k* thin film thicknesses for 150°C, 200°C, 250°C, 300°C, and 350°C are 51, 43, 50, 31, and 44 nm, respectively, from spectroscopic ellipsometry. The SiO_2_ interfacial layer thickness is approximately 1.6 nm, which leads to much larger capacitance than the high-*k* thin film. Thus, lossy interfacial layer effect is excluded for the cerium oxide samples. In terms of part 3, the surface roughness is not responsible for the observed extrinsic frequency dispersion of the high-*k* thin films used in the paper. With respect to part 4, the poly depletion effect will become more significant leading to reduced surface potential, channel current, and gate capacitance. However, the polysilicon depletion effect is not under consideration for the samples here because the gates of the MOS capacitor samples were Au-fabricated by thermal evaporation through a shadow mask. Finally, as regards part 5, for oxide thickness down towards 1 to 3 nm, the quantum mechanical effect should be taken into account. The cerium oxide samples are not suitable for the domain (greater than 30 nm at least). All in all, after taking into account all extrinsic causes of the frequency dispersion mentioned above, the intrinsic effect (dielectric relaxation) of the high-*k* dielectric thin films is proved to be the main impact factor for the *C*-*V* frequency dispersion observed in Figure 4. Similar observations are also seen in the rest of the as-deposited samples (deposition temperatures from 150°C to 350°C). At the frequency of 1 MHz, the capacitance is 300 pF in strong accumulation. Enhanced capacitance (1,420 pF) in strong accumulation at a frequency of 100 Hz is observed, which is more than four times the capacitance measured at 1 MHz. Moreover, it is found that the value of accumulation capacitance is inversely proportional to the frequency. The *C*-*V* measurements of the annealed samples (solid lines) are also shown in Figure 4. In contrast to the as-deposited high-*k* thin films, the annealed samples show a pronounced accumulation capacitance reduction, which is mainly due to the increased interfacial layer (IL). One kind of high-*k* materials were researched by our group before: La-doped ZrO_2_ films, with a thickness of 35 nm deposited on *n*-type Si(100) substrates by liquid injection ALD at 300°C [14]. The 35-nm-thick La_0.35_Zr_0.65_O_2_ layers retained their thickness after PDA, but the IL (SiO_*x*_) increased from 1.5 nm on the as-deposited samples to 4.5 nm after PDA at 900°C in N_2_, respectively, which is attributed to either an internal or external oxidation mechanism. As high-*k* layer is on the top of the IL, the capacitance of high-*k* layer is in series of the IL capacitance. When the thickness of the IL is increased, the capacitance of the IL is decreased, and it is no longer much larger than the high-*k* layer capacitance. Therefore, the total capacitance (including the capacitance of the high-*k* layer and the IL capacitance) is decreased significantly. Generally speaking, the most obvious effect of annealing is therefore to weaken the accumulation capacitance and hence reduce the *k*-value. Insignificant frequency dispersion is observed from 100 Hz to 1 MHz. The annealed capacitance of 100 Hz decreases by approximately 70% of the as-deposited sample. The accumulation capacitance value is 410 pF below 100 Hz. The capacitances from 1 kHz to 1 MHz are in the range of 180 to 240 pF.

In order to further investigate the frequency dispersion for CeO_2_, a normalized dielectric constant (to the dielectric constant at 100 Hz) is utilized to quantitatively characterize the dielectric constant variation. At the start, both as-deposited and annealed samples are used. Concerning the 250°C samples, the comparison between the as-deposited and annealed is given in Figure 5. It is observed that the dielectric relaxation for the as-deposited sample (triangle symbol) is much pronounced than that of the annealed one (square symbol). Within the range of various frequencies, the normalized *k* value of the as-deposited sample is lower. Obviously, the worst-case situation occurs at 1 MHz when the normalized dielectric constant is 0.11. It means that the *k* value is only 10% of the highest *k* value measured below 100 Hz. The conclusion is made from the data that the frequency dispersion for the CeO_2_ samples has been alleviated after annealing. From the analysis of Figure 2, the grain size for annealed samples is larger than the as-deposited one. It is easy to make an inference that grain size affects dielectric relaxation. The smaller grain size has a more intense dielectric relaxation. These findings are in good agreement with the theoretical and experimental studies proposed by Yu et al. [18], which reported the effect of grain size on the ferroelectric relaxor behavior in CaCu_3_TiO_12_ (CCTO) ceramics. Since its unusual dielectric properties were discovered in 2000, an ABO_3_-type perovskite material, CCTO, in which Ca^2+^ and Cu^2+^ share the A site, has attracted extensive attention. Many mechanisms have been proposed to interpret the nature of its giant dielectric response, and the frequency dispersion of the CCTO samples is found to be dependent on grain size. Thus, it is considered to be the supporting evidence of the cerium oxides. The response for the normalized dielectric constant values of CCTO over different frequencies (100 Hz and 1, 10, and 100 kHz) is extracted and shown in the inset of Figure 5. In the inset, the CCTO ceramics have different grain sizes (small, medium, and large). Strong frequency dispersion for all the samples with different grain sizes is related to the frequency-dependent boardening and shift of glasslike transition temperature. It is associated with the slowing down of dipolar fluctuations within the polar nanodomains. The dielectric relaxation for the small grain size sample is the worst case. The dielectric constant of 100 kHz is only 10% of the value below 100 Hz, which is similar to the as-deposited 250°C CeO_2_ sample. The medium-grain-size CCTO sample is superior to the small-grain-size sample within the range of various frequencies. Moreover, the large-grain-size sample performs better than the medium-sized one. The effect of grain size mainly originates from higher surface stress in smaller grain due to its higher concentration of grain boundaries. To illustrate this point, surface stress in the grains is high, medium, and low for the small-, medium-, and large-grain-size CCTO samples, respectively. As surface stress increases, the glasslike transition temperature decreases considerably. This is attributed to the enhancement of the correlations among the polar nanodomains. Ultimately, both frequency dispersion and relaxation strength, as typical characteristic of relaxor ferroelectrics, will increase when grain sizes decrease.

Figure 6 shows the normalized dielectric constants for all the as-deposited CeO_2_ samples under the different deposition temperatures (150°C, 200°C, 250°C, 300°C, and 350°C). It is known from the XRD (Figure 1, inset) and Raman spectra (Figure 3) that grain size increases as the deposition temperature increases. The relationship between the grain size value and the deposition temperature is shown as follows: 6.13 nm for 150°C, 6.69 nm for 200°C, 8.83 nm for 250°C, 15.85 nm for 300°C, and 23.62 nm for 350°C. Large dielectric relaxation is observed for the sample of 6.13 nm (diamond symbol). The minimum *k* value at 1 MHz is one third of the maximum value at 100 Hz. When the deposition temperature increases, the dielectric relaxation is even worse for the sample of 6.69 nm (square symbol). The *k* value variation is more significant across all the frequency range. In addition, the most severe dielectric relaxation is measured for the sample of 8.83 nm (star symbol). The worst situation is that the *k* value calculated at 1 MHz is only 10% of the *k* value below 100 Hz. Also, from the preceding figure, the normalized dielectric constants are the smallest for all of the frequencies, which means that the dielectric constant makes the most significant value drop within the region of different frequencies for the sample of 8.83 nm. The sample of 15.85 nm (triangle symbol) has significant improvement on dielectric relaxation. The *k* value variation from 100 Hz to 1 MHz is narrowed accordingly. The sample of 23.62 nm (round symbol) shows a more stable frequency response. As a consequence, it is not always true for the inference we made earlier: the smaller grain size has a larger dielectric relaxation (the sample of 8.83 nm has the worst dielectric relaxation, but 8.83 nm is not the smallest grain size value among all the samples). Nevertheless, if a comparison is made between samples of 6.13 nm (the smallest) and 23.62 nm (the largest), the larger-grain-size sample is shown to have better dielectric relaxation performance. It is also consistent with our previous experimental results [9]. However, the trade-off for the 23.62-nm sample is that the dielectric constant is smaller than the 6.13-nm sample. Especially in terms of the dielectric constant, on 100 Hz, the dielectric constant for the 23.62-nm sample is only half of the value for the 6.13-nm sample. Moreover, in 1 MHz, the dielectric constant for the 23.62-nm sample is two thirds that of the value for the 6.13-nm sample. Thus, the 23.62-nm samples perform best at the expense of the dielectric constant. Similarly, the effect of grain size on dielectric relaxation is found on the Nd-doped Pb_1-3*x*/2_Nd_*x*_(Zr_0.65_Ti_0.35_)O_3_ composition (PNZT) [19], where *x* = 0.00, 0.01, 0.03, 0.05, 0.07, and 0.09, respectively. Lead-based perovskite ferroelectric ceramics are widely applied in multilayer capacitors, microelectromechanical systems, and integrated devices such as ferroelectric memories, infrared sensors, microactuators, etc. Moreover, lead zirconium titanate is one of the best lead-based materials that have been studied extensively recently. The PNZT samples were fabricated according to the A-site vacancy formula and were prepared by the traditional mixed-oxide solid-state reaction method. The grain size decreases as Nd doping (*x*) increases. The normalized dielectric constant vs. frequency is extracted and shown in the inset of Figure 6. Strong frequency dispersion is observed for all of the samples. It is clear that the deteriorative degree of dielectric relaxation increases from 12.1 nm, reaches the peak at 22.5 nm, and then declines. A comparison between the samples of 12.1 and 25 nm is made. Uniformly, the sample with the grain size of 25 nm is shown to perform superior on dielectric relaxation. The dielectric constant frequency response of the PNZT samples shares exactly the same response for the CeO_2_ samples (one dielectric relaxation peak within the frequency range). A possible reason [19] to the cited observation could be the broadened dielectric peak and the transition temperature shift. The dielectric constant shows phase transition as expected for normal ferroelectrics. The region around the dielectric peak is broadened, which is one of the most important characteristics of disordered perovskite structure with the diffuse phase transition. The transition temperature is found to shift forward to lower temperature with the grain size from 12.1 to 22.5 nm, while the transition temperature remains at the same position with further increasing grain size. Concerning the strong frequency dispersion, it is mainly attributed to the low-frequency space charge accumulation effect. Such strong frequency dispersion in dielectric constant appears to be a common feature in ferroelectrics associated with non-negligible ionic conductivity. Therefore, the reason for the dielectric relaxation of the PNZT samples could be the possible mechanism behind the frequency dependence of the *k* value of the CeO_2_ samples.

Many dielectric relaxation models (Cole-Davidson, Havriliak-Negami, and Kohlrausch-Williams-Watts) were proposed to interpret the dielectric relaxation, which is also termed as the frequency dependence of the *k* value. The Havriliak-Negami (HN) model is suitable for almost all of the high-*k* materials as it has three parameters for fitting (*α*, *β*, and *τ*). In contrast, the Cole-Davidson (CD) model only has two parameters for fitting (*β* and *τ*). Thus, if the CD model is able to fit the cerium oxides, it will be more significant for the specified physical mechanism compared to the HN model. Concerning the Kohlrausch-Williams-Watts (KWW) model, it has also two adjusting parameters for fitting (*β* and *τ*). The CD and KWW models have certain links in both high frequency and low frequency approximations. Besides, the CD model is widely used in glass-forming materials to explain the frequency dependence of the dielectric constants [20]. Here, dielectric relaxation can be described by the CD law for all of the CeO_2_ samples. CD fittings are denoted by solid lines in Figure 6. In 1951, D. W. Davidson and R. H. Cole [21] proposed the CD equation to interpret data observed on propylene glycol and glycerol based on the Debye expression. The CD equation can be represented by ϵ*(*ω*).

(1) $$\epsilon^{*}\left(\omega \right)=\frac{\epsilon_{\infty }+\left(\epsilon_{s}-\epsilon_{\infty }\right)}{{\left(1+\mathrm{i\omega \tau}\right)}^{\beta }}$$

where *β* controls the width of the distribution (0 ≤ *β* ≤ 1). *β* = 1 is for the Debye relaxation. For angular frequencies *ω* = *2πf* >*1*/*τ*, the CD model exhibits an asymmetric broadening of the spectrum towards high *f*[22]. The real part (the *k* value) and imaginary part of Equation (1) are given:

(2) $$\epsilon^{'}\left(\omega \right)=\epsilon_{\infty }+\left(\epsilon_{s}-\epsilon_{\infty }\right){\left(\cos\varphi \right)}^{\beta }\cos\beta \varphi$$

(3) $$\epsilon^{''}\left(\omega \right)=\left(\epsilon_{s}-\epsilon_{\infty }\right){\left(\cos\varphi \right)}^{\beta }\sin\beta \varphi$$

(4) $$\varphi =\tan^{-1}\left(\omega \tau \right)$$

As-deposited and annealed cerium samples are fitted with the CD model in Figure 5. The fitting parameters of the CD equation, beta (*β*) and tau (*τ*), are listed as follow: beta for the as-deposited sample is 0.22, and the value for the annealed sample is 0.15. In the mean time, tau for the as-deposited sample is 0.00082, and the value for the annealed sample is 0.00089. The values for both samples are quite close. The fitting parameters of the CD equation, *β* and *τ*, are shown in Figure 7. The left *Y* coordinate axis is for the beta value, and the right *Y* coordinate axis is for the tau value. The *X* coordinate axis is for the grain size of the samples. It is clear that the trend of beta increases from 6.13 nm, peaks at 8.83 nm with the beta value of 0.21, and then descends. Thus, the curve of beta is found to be consistent with a deteriorative degree of dielectric relaxation, which agrees with the fact that the slope of the real part *ϵ*' to the frequency is dependent on the parameter beta. To be more specific, the deteriorative degree of dielectric relaxation is shown to be consistent with the beta value from the CD modeling by quantization, which is due to the beta-dominating exponent part in CD modeling (Equation 2). Hence, to a certain extent, beta value represents the deteriorative degree of dielectric relaxation. Furthermore, the asymmetry of the loss factor is more serious as the parameter beta increases. Concerning the parameter tau, the trend decreases from 6.13 to 23.62 nm. The real part of the CD equation shifts horizontally to higher frequency value as the values of tau decrease. Usually, tau is identical in form with the Vogel-Fulcher-Tammann (VFT) law for the temperature dependence of viscosity of a number of polar materials [23]. Viscous flow in amorphous glass-forming materials is a thermally activated process. According to the experimental data, follows the VFT law. The VFT law is given as follows:

(5) τ=A·eEakT,

where *E*_a_ is the activation energy of ion transport over the entire temperature range, *T* is a characteristic temperature corresponding to the freezing temperature of the material within VFT approach, *k* is the Boltzmann constant, and *A* is approximately a constant. The origin of the VFT law is the increase of the range of elastic interaction between local relaxation events. The transition of glass-forming materials on lowering the temperature may appear conceptually simple, yet this phenomenon has turned out to be one of the most difficult and controversial problems in condensed matter physics, the problem of the glass transition. At high temperature, relaxation time *τ* follows an Arrhenius dependence. Upon lowering the temperature, *τ* almost universally deviates from an Arrhenius dependence and follows the VFT law. Through the VFT law, the activation energy *E*_a_ and the freezing temperature *T* can be obtained. Tau *τ* is probably determined by the as-deposited temperature. So, the related activation and freezing temperature could be calculated afterwards. The cause of distribution of the relaxation times has been associated with certain particular factors, e.g., the suggestion made by Kliem and Arlt [24] concerning the occurrence of protonic resonance and Cabeza et al. [25] concerning the porosity effect. Equally, dielectric relaxation of the PNZT samples can be modeled by the CD law as well. The inset of Figure 7 shows the relationship between the CD fitting parameters (beta and tau) and the grain size value likewise. The trend of beta rises from 12.1 nm, peaks at 22.5 nm with the beta value of 0.03, and then glides back downwards within the range of 22.5 to 25 nm. The beta value is therefore shown to represent the deteriorative degree of dielectric relaxation. In the same manner, the trend of tau decreases from 12.1 to 25 nm. The trend of beta and tau for the PNZT samples is similar to the trends observed for the CeO_2_ samples.

## Conclusions

The ALD CeO_2_ samples were grown as crystalline thin films for a range of substrate temperatures within the ALD growth window of the Ce[mmp]_4_ precursor, with water as an oxidant. XRD and Raman spectra show an increase in grain size for increasing growth temperatures. From the *C*-*V* measurement of the samples, strong frequency dispersion is observed. In order to further investigate the dielectric relaxation, the normalized dielectric constant is utilized for the CeO_2_ samples of different grain sizes. The CeO_2_ samples have better dielectric relaxation behavior after annealing since the annealed samples have a larger grain size. Within the grain size range of the CeO_2_ samples (6.13 to 23.62 nm), the most serious frequency dependence of the *k* value is found in the sample of thickness 8.83 nm. A similar relationship between grain size and dielectric relaxation is also observed in CCTO and Nd-doped PNZT samples. The mechanism of grain size effects is attributed to the alignment enhancement of the polar nanodomains.

## Competing interests

The authors declare that they have no competing interests.

## Authors' contributions

CZ extracted the data and drafted the manuscript. CZZ led the experiment and supervised the project. MW prepared the samples and performed the characterization. ST and PC participated in the discussions. PK completed the measurement. All of the authors read and approved the final manuscript.

## Authors' information

CZ is a PhD student in the University of Liverpool. CZZ is a professor in Xi'an Jiaotong-Liverpool University. MW is a research associate in the University of Liverpool. ST and PC are professors in the University of Liverpool. PK is a research fellow in the University of Liverpool.
